# Electrocardiographic and Echocardiographic Parameters in Pega Breed Donkeys: A Descriptive Study

**DOI:** 10.3390/ani13050861

**Published:** 2023-02-27

**Authors:** Amanda Sarita Cruz-Aleixo, Karina Cristina de Oliveira, Lucas Vinícius de Oliveira Ferreira, Dario Alejandro Cedeo Quevedo, Raíssa Karolliny Salgueiro Cruz, Miriam Harumi Tsunemi, Simone Biagio Chiacchio, Maria Lucia Gomes Lourenço

**Affiliations:** 1 School of Veterinary Medicine and Animal Science, Department of Animal Health, São Paulo State University (UNESP), Botucatu 18618-681, Brazil; 2 Department of Animal Health, University of Nariño, Pasto 7311449, Colombia; 3 Department of Animal Health, Cesmac University Center, Maceió 57051-160, Brazil; 4 Institute of Biosciences, São Paulo State University (UNESP), Botucatu 18618-970, Brazil

**Keywords:** heart, equine, electrocardiogram, echocardiogram, donkeys

## Abstract

**Simple Summary:**

Cardiovascular parameters of donkeys are scarce in the literature. The aim of the present study was to describe the echocardiographic and electrocardiographic parameters in donkeys to contribute to new studies on the species. Fifty Pega breed donkeys were evaluated. Echocardiographic and electrocardiographic evaluation is feasible in donkeys. For the analysis and investigation of cardiovascular diseases in the species, it is necessary to evaluate specific parameters for donkeys. Anatomical and physiological differences have been reported between donkeys and horses; therefore, the use of clinical data, treatments, and diagnostic protocols from horses to donkeys can trigger diagnostic errors and inappropriate therapeutic administration.

**Abstract:**

Clinical, electrocardiographic and echocardiographic parameters in Pega donkeys are scarce in the literature; hence, this study was performed to describe the echocardiographic and electrocardiographic measurements in Pega breed donkeys. The objectives of this study were to describe and illustrate the clinical, electrocardiographic, and echocardiographic parameters in Pega donkeys used for reproduction. Fifty Pega breed donkeys were evaluated, with an average age of 3.4 years and with 20 males and 30 females. In each animal, the electrocardiographic examination at rest was performed using the TEB^®^ computerized system, and the echocardiographic examination was performed using an ultrasound device with a Doppler function multifrequency sectorial transducer in 2D mode (Sonosite^®^ M turbo). Standardizing the electrocardiographic and echocardiographic parameters for the Pega breed donkey can contribute to future assessments regarding possible changes that excessive effort can promote in these parameters to a management engrossed on animal welfare.

## 1. Introduction

Currently, donkeys are animals of great economic interest and as companion animals, a fact that has increased awareness of the welfare and care of these animals, increasing the demand for specialized veterinary services [1]. Anatomical and physiological differences have been reported between donkeys and horses; therefore, the use of clinical data, treatments, and diagnostic protocols from horses to donkeys can trigger diagnostic errors and inappropriate therapeutic administration [2]. There are a variety of differences in behavior and social organization between donkeys and horses, although they are often housed as companion animals or as a homogeneous group. However, this does not mean that the nature of social relationships between different species of equids is the same as that between their own species [3].

Brazil has the largest herd of horses in Latin America and the third largest in the world. There are 8 million donkeys and horses, with transactions of R$ 7.3 billion only through the production of horses [4]. The main breeds of donkeys used to obtain mules are the Paulista or Brasileiro, the Pega, and the Northeastern donkey. The Pega is a national donkey breed with economic value throughout the national territory, and it is raised for the production of new breeders and for marching mules. It is a versatile animal used for pack animals, in preparing the soil, herding cattle, horseback riding, functional tests, walking contests among many modalities, and is currently the most used in the training of saddle-type. The animals of the Pega breed are strictly gaiters and have great value in the production of hybrids (mules). The magpie donkey market is focused on breeding. The Pêga donkey is medium-sized with a minimum height at the withers of 125 cm for males and 120 cm for females. Male animals of this breed weigh approximately 300 kg, and females weigh 240 kg [5].

A suspicion of potential heart disease may appear after routine examinations or during the prepurchase evaluation of an animal. On the other hand, the care of an equine may be requested due to a specific complaint of cardiovascular signs; however, this assessment is not as widespread in donkeys [6]. The clinical symptoms or the absence of them are important factors in the assessment of a possible cardiac study for reflection on the potential differential diagnoses [3]. Heart disease is rare in horses compared to other domestic species due to the large cardiac reserve, and overt clinical signs are often seen only when there is severe dysfunction or during forceful exercise. Although murmurs and arrhythmias are commonly detected in equines, they are often of physiological origin and have no pathological significance [7].

Arrhythmias during or immediately after exercise are common occurrences in equine athletes. The spectrum of these rhythm variations covers clinically irrelevant arrhythmias, arrhythmias that can cause poor performance, and rhythms with risk of death [8]. A standard base-apex electrocardiogram at rest should be performed in all horses with arrhythmia not attributable to second-degree atrioventricular block [9]. Digital electrocardiogram telemetry systems are readily portable and can be used to obtain real-time digital monitoring and recording at rest or during exercise [10]. In donkeys, there is little information in the literature regarding arrhythmic events that may occur during exercise, and there is no standardized information about possible arrhythmias that can be considered common in the species [11,12].

Exercise-induced cardiac fatigue and cardiac dysrhythmias are well-described conditions identified in high-level human athletes that increase in frequency with intensity and duration of exercise [13,14]. Thus, since electrocardiographic and echocardiographic parameters in Pega donkeys are scarce in the literature, as well as the description of possible arrhythmic events that may be considered common in the species and the description of cardiovascular diseases, the objectives of this study were to describe and illustrate the clinical, electrocardiographic at rest and echocardiographic parameters in Pega donkeys, which may contribute to future studies aiming to assess the influence of exercise on such parameters as well as the activity of the autonomic nervous system in these animals during overexertion since they are already adapted for such efforts.

The description of these parameters in Pega donkeys can contribute to a better understanding of specific changes that may occur in donkeys, where evaluations in horses are often used as a reference for these animals, which can lead to erroneous conclusions, and the particularities of different categories and breeds of animals must be respected.

## 2. Materials and Methods

### 2.1. Animals and Study Site

The present study was carried out according to animal welfare standards and approved by the Ethics Committee on the Use of Animals (CEUA) of the Faculty of Veterinary Medicine and Animal Science of the São Paulo State University “Júlio de Mesquita Filho”, Botucatu Campus, under the protocol CEUA-0029/2021.

The present study was carried out on 50 clinically healthy adult donkeys aged under 13 years, 20 males (stallions) and 30 females. Before enrollment in the study, all donkeys underwent complete physical examination to exclude systemic conditions (colic, respiratory diseases, orthopedic diseases).

The animals were kept in paddocks and individual pens and were fed with Tifton hay produced on the property and pelleted feed with 15% protein; they were offered a total of 3 kg of daily feed divided into two portions, and commercial mineral salt was added for horses at will.

The study was carried out at the stud farm Criatório Ximbó, located in the district of Maristela-SP, belonging to the city of Laranjal Paulista-SP, with latitude 23°02′9″ south and longitude 47°50′12″ west. For this purpose, 50 donkeys were evaluated, with an average age of 3.4 years; there were 20 males and 30 females, all from the same establishment.

**Figure 1:**
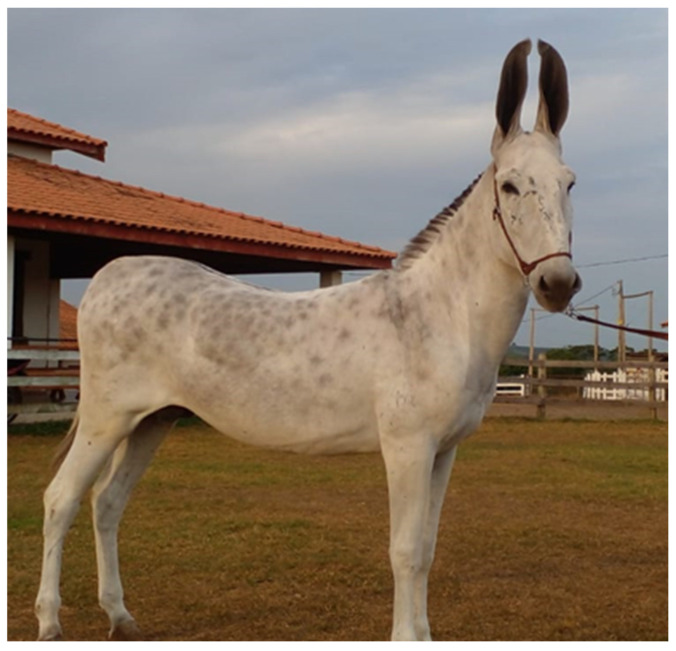
A donkey Pega breed, 8 years old, male.

The average height at the withers of the animals was 1.30 m, and the average weight was 230 kg (the males had a height at the withers of approximately 1.35 m and the females 1.20 m, with the weight of the males being approximately 260 kg and the females 230 kg). The animals in the present study were mainly used for reproduction. Figure 1 illustrates a donkey, Pega breed, 8 years old, male.

The study was carried out after approval from the animal use ethics committee in accordance with the animal welfare standards upon signature of the owner’s informed consent form.

### 2.2. Analysis Groups

The animals were evaluated according to their clinical parameters, such as heart rate (HR—beats per minute—bpm), respiratory rate (RR—movements per minute—mpm), mucosal color, rectal temperature (°C), and capillary filling time (CFT).

As there are no specific heart rate (HR) parameters for donkeys, the HR values were divided according to the reference for horses (28–60) and similar to the division into groups according to HR adopted by Guccione and colleagues [12]. Thus, the division of the study according to HR by class was as follows: class 1: HR ≤ 30 bpm, class 2: HR 30–60 bpm, and class 3 > 60 bpm.

### 2.3. Electrocardiographic Examination

The electrocardiographic examination was performed at rest using the TEB^®^ computerized system (Brazilian Electronic Technology, São Paulo-SP, Brazil), and the electrocardiographic tracings were recorded with a sensitivity of 1 mV = 1 cm and at a speed of 25 mm/s, compiling the bipolar I, II, and III lead and unipolar amplified aVR, aVF, and aVL.

The electrodes were attached to the skin using “alligator” clips soaked with alcohol. Recordings were performed as described by Loon and colleagues (2010) [13], placing the positive electrode on the left side above the heart apex (green), just behind the olecranon; the negative electrode on the right side, cranial to the scapula, close to the jugular vein (red); and the ground electrode attached to the animal’s withers (black). For each electrocardiographic record, HR, rhythm, P wave duration and amplitude, PR interval duration, QRS complex duration, R wave amplitude, S wave amplitude, QT interval, and QTc duration were analyzed, as well as the duration and amplitude of the T wave.

### 2.4. Echocardiographic Examination

To obtain the echocardiographic evaluation, the animals were kept in a station, manually contained, without any type of sedation for the examination. Trichotomy and cleaning of the right and left thoracic regions were performed 10 cm above the height of the olecranon, with the right thoracic limb carefully placed forward.

The echocardiographic examination was performed using an ultrasound device (M-turbo Sonosite model, Fujifilm do Brasil Ltd.a., São Paulo-SP, Brazil) with a Doppler function and a 2–8 MHz multifrequency sectorial transducer in 2D mode. The transducer was positioned between the 4th and 5th intercostal space. The measurements were always performed by the same operator, obtaining three measurements from each assessment.

Through the right parasternal window, in cross-section, M-mode, at the height of the papillary plane, in diastole were measured: interventricular septal thickening (IVS), left ventricular internal diameter (LVID), left ventricular free wall thickness LVFW, and right ventricular diameter in diastole (RIVDd). In systole, interventricular septal thickening (IVS), left ventricular internal diameter (LVID), and left ventricular free wall thickness (LVFW) were analyzed.

The fractional shortening of the left ventricle (FS) was obtained with the Teichholz method. To calculate the FS (%), the following formula was used: (LVIDd–LVIDs/LVIDd) × 100, and the left ventricular ejection fraction (EF) was also recorded.

**Figure 2:**
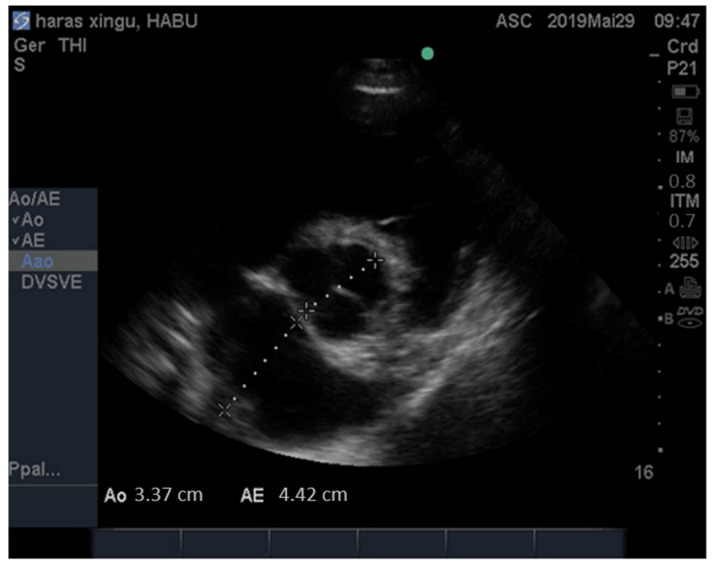
Image obtained by the two-dimensional echocardiogram of a donkey, male, 6 years old, right parasternal window, cross-section, aortic plane, left atrium/aorta ratio (LA/Ao).

The diameter of the left atrium (LA) and the aorta on the same plane was measured, and subsequently, the left atrium/aorta ratio was calculated (LA/Ao) (Figure 2). Pulmonary flow velocity (pulmonary velocity) and pressure gradient between the right ventricle and pulmonary artery at the level of the pulmonary plane was also measured.

Pulmonary diameter and aortic diameter were also measured, and the pulmonary/aorta ratio (pul/ao) was obtained from the cross-section of the base. Aortic flow velocity (Ao) was measured. The pressure gradient between the left ventricle and aorta artery was also compiled by the left parasternal window to optimize the outflow tract view.

### 2.5. Statistical Analysis

The results are illustrated with the mean, standard deviation, and minimum and maximum values. To analyze the parameters, the normality test used was the Shapiro–Wilks test; to compare the proposed moments, the Mann–Whitney test was used. All discussions were carried out at a 5% significance level.

## 3. Results

The mean, standard deviation, and minimum and maximum values of the clinical parameters in the Pega donkey are represented in Table 1. The average age of the animals was 3.4 years, with an HR of 67 bpm and an RR of 35 mpm.

The mean values and standard deviation of echocardiographic measurements and parameters of the healthy Pega breed donkeys are represented in Table 2.

The mean values and standard deviation of electrocardiographic measurements and parameters of the 50 healthy Pega breed donkeys are tabulated in Table 3.

**Figure 3:**
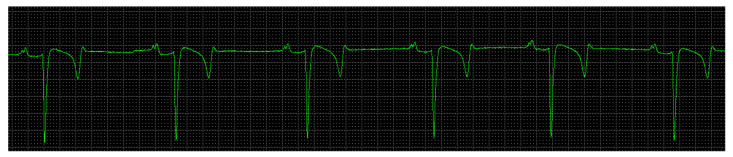
Electrocardiogram, donkey, male, 6 years old, base-apex plane, rS pattern and bifid P waves.

Table 3 shows the mean HR of 50 donkeys (65 bpm). According to the class division approved in the study, no animal presented sinus bradycardia and an HR below 35 bpm (class 1 = 0), 22 animals (44%) had an HR within class 2 (30–60 bpm), and 28 animals (56%) presented an HR compatible with class 3 (>60 bpm). In view of the horse reference, the predominant rhythm was sinus tachycardia. Bifid P waves were observed in the animals in the present study. There was a predominance of the QRS complex pattern of the rS-type (Figure 3).

The amplitude of the T wave varied, with 45% (22 animals) showing positive T waves, 28% (14 animals) showing negative T waves, and 28% showing biphasic T waves. Arrhythmic events were not detected in the animals in the present study.

## 4. Discussion

The present study represented the normal reference range of echocardiographic and electrocardiographic measurements and parameters in healthy Pega donkeys. The mean HR results in this study from a clinical examination (67 bpm) and electrocardiogram (ECG) (65 bpm) were higher than those reported by Guccione and colleagues [12] in donkeys (47 bpm). The authors evaluated the HR in 15 donkeys with the Holter examination during the day and night. The mean HR in our study and that reported by the mentioned authors in horses are superior, indicating that there are differences in the activity of the autonomic nervous system (ANS) between donkeys, asses, and horses. There is a need for studies explaining and comparing ANS activity between them. The different acquisition techniques of these parameters should also be considered since in our study, the HR recording was immediate to manipulation, which may have contributed to higher values.

Escudero and colleagues [14] carried out a study to evaluate the electrocardiographic parameters in both in Zamorano-Leones using the conventional method. In their study, most animals presented a bifid P wave similar to what occurred in our study. In our study, there was a predominance of the QRS complex pattern of the rS-type, differing from the study by the aforementioned authors, who found predominance of the QR pattern. We believe that these differences may be due to different breeds and sexes, as the authors mention that they found a QS pattern only in females. A negative shape was present for the T wave in lead II, which is in agreement with the results obtained by Escudero and colleagues.

The method used for the electrocardiographic assessment and the breed may have influenced the different results for the duration and amplitude of the electrocardiographic waves. Al-Haidar and colleagues [15] demonstrated in horse studies that breed has an influence on echocardiographic parameters. The scarce information in the literature on electrocardiographic parameters in donkeys requires studies aimed at standardizing clinical and electrocardiographic parameters with a focus on breed differences and not only in relation to the difference between species. Just as in horses, there are differences in echocardiographic parameters between breeds; in donkeys, there may be differences in electrocardiographic parameters.

The echocardiographic parameters obtained in the M mode in the present study were higher when compared with the donkey parameters originated by Roberts and colleagues [16], and LVFWd for the Pega breed was similar to those of the aforementioned authors for males with an average of 1.68 cm.

Horse heart size and dimensions increase in relation to the animal’s height and weight, but according to Farag and colleagues [17], a study with donkeys did not find an association between echocardiographic parameters in both the B mode and the M mode with age and body weight. In our study, we did not divide the animals according to weight, but the parameters analyzed as a whole are for males and females. In horses, age has a great effect on the inner diameter of the aortic and pulmonary arteries. In addition, body weight also has a significant effect on all echocardiographic dimensions, but sex has no effect on any of them [15]. In humans, the aortic diameter may increase with age, and collagen changes and systemic arterial hypertension may contribute to aortic artery aneurysm [18,19]. Weight, as already proven in allometric scales, influences echocardiographic parameters. Therefore, studies dividing Pega breed donkeys according to different age groups and body weights are needed.

Scarce data in the literature and the need for racial distinction emphasize the need to standardize electrocardiographic and echocardiographic parameters in donkeys. Because the animals do not show obvious clinical signs, cardiovascular diseases in this species can be underdiagnosed. The early diagnosis of changes in heart rate and cardiovascular structure can contribute to the use of these animals in modalities that require excessive effort and reduce the number of conditions that go undiagnosed [20].

Cardiovascular disease can be seen in donkeys, presumably with a prevalence similar to horses. Available information about these conditions is uncommon in this species, which does not make the diseases rare. However, a large proportion of cardiovascular diseases are described in horses that lead to reduced performance. Considering that donkeys and assess are not commonly used as riding animals and their athletic attitude is limited, these diseases could easily be underdiagnosed [16].

According to Reef and colleagues [21], a regular sinus rhythm with a range HR at rest from 24 bpm to 44 bpm is the most common rhythm detected in horses. The adult horse has high vagal tone present at rest, especially in good physical conditions, resulting in low resting heart rates and rhythm disturbances that disappear with excitement, exercise, or any intervention that increases the sympathetic tone. Thus, according to reports by Reef and colleagues [21] and with the mean heart rates found in the animals in this study, we observed that donkeys have high HR compared to horses, which may lead us to infer that reference values for horses should not be used to assess normality parameters for donkeys. Studies using HRV analysis in this context are necessary since, as illustrated in this study, donkeys seem to have a higher sympathetic tone when compared to horses, which have a predominance of vagal tone.

Several studies describe cardiovascular affections in horses, as well as electrocardiographic patterns [22,23]; echocardiographic patterns in the species [24,25,26] and in foals [27] are also related to weight, sex, and age, as well as common changes in the category of athlete animals [21]. According to the echocardiographic parameters obtained in the present study, donkeys seem to have lower values for these parameters in horses, as described in the literature.

Because Pega donkeys are widely used in work modalities that require physical effort [28], we highly recommend echocardiographic and electrocardiographic parameter examinations. In recent years, in human and veterinary medicine, modern Doppler echocardiography techniques have been introduced to assess myocardial function by measuring cardiac muscle velocities and deformations. Studies in marathon runners on the effect of training on myocardial deformation parameters demonstrated that longitudinal myocardial contractility and high early diastolic velocities (E wave) were directly correlated with increased left ventricular diastolic internal diameters [29,30]. Future studies in donkeys can also be used to assess myocardial function to establish the impact that high workloads can have on the cardiovascular system.

Finally, several studies have illustrated the effect of estrogen and testosterone on ventricular function. Castration studies also suggest gender-specific differences in cardiac structure and function. Castrated male rats showed reduced heart weight with a significantly lower ejection fraction and cardiac output, hypocontractility, and delayed cardiomyocyte relaxation, whereas exercise attenuated and testosterone replacement completely reversed these effects. Male rats showed decreased contractile reserve and a faster transition to heart failure with left ventricular dilatation, loss of concentric remodeling, and diastolic dysfunction compared to their female counterparts. In addition, estradiol is positively associated with right ventricular ejection fraction while in men and women, testosterone was positively associated with right ventricle mass and volume [31]. We believe that the effects of estrogen may have influenced our results, and studies also focusing on hormone dosages could contribute in greater magnitude to illustrate the effect of hormones in donkeys on electrocardiographic and echocardiographic parameters.

Heart rate variability corresponds to the fluctuation in the interbeat intervals and can measure the functioning of the ANS. Actually, in horses, as well as in other animals, HRV analysis methodologies and data interpretation are still debated. Therefore, it is interesting to investigate the use of HR and HRV in donkey clinics and research, especially in relation to animal welfare, where noninvasive and reliable indicators are urgently needed [32]. Our study may contribute to future studies aiming to evaluate ANS activity in donkeys.

Our study has some limitations. The age of the animals may have influenced the results since the groups included animals aged 13 years and 1 year. We used animals used only for reproduction. Studies using other categories of animals are needed. We believe that gender influenced our results, but studies using hormone levels could provide better information. We did not divide the animals in the group into different types of weight, which may have influenced the results because males tend to have higher weight than females, and we used animals of both sexes in our group.

## 5. Conclusions

Echocardiographic and electrocardiographic evaluation is feasible in donkeys, and there are parameter similarities described in the equine species. For the analysis and investigation of cardiovascular diseases in the species, it is necessary to evaluate specific parameters for donkeys. The standardization of such parameters can contribute to future studies aiming to evaluate the influence of exercise on the parameters of this species since, in horses, the changes induced by exercise in cardiac electrical conduction are described in the literature, as well as contributing to studies evaluating the heart rate variability in the species.

## Figures and Tables

**Table 1:** Clinical parameters (mean, standard deviation, minimum and maximum) in Pega breed donkeys.

Parameter	Mean ± SD	Minimum	Maximum
Age (years)	3.46 ± 3.08	1.00	13.00
HR (bpm)	67.20 ± 16.38	40.00	120.00
RR (mpm)	35.60 ± 9.08	20.00	60.00
T °C	37.82 ± 0.63	36.60	39.10

**Table 2:** Echocardiographic parameters in Pega breed donkeys from the M-mode of the LV and the short axis view of the LA and aorta (mean, standard deviation, minimum and maximum).

Parameter	Mean ± SD	Minimum	Maximum
IVSd (cm)	1.74 ± 0.34	1.26	2.59
LVIDd (cm)	6.80 ± 1.08	4.60	9.17
LVFWd (cm)	1.73 ± 0.33	1.17	2.67
IVSs (cm)	3.05 ± 0.55	1.83	4.59
LVIDs (cm)	4.07 ± 0.71	2.45	5.47
LVFWs (cm)	2.59 ± 0.46	1.77	3.84
RIVDd (cm)	1.81 ± 0,61	1.0	3.74
EF (%)	68.82 ± 7.09	53.00	86.00
FS (%)	39.92 ± 5.99	27.90	55.80
LA (cm)	5.27 ± 0.64	4.03	6.51
Ao (cm)	4.03 ± 0.59	2.67	5.14
LA/Ao	1.31 ± 0.14	1.02	1.67
Pulmonary diameter	3.28 ± 0.45	2.25	4.30
Aortic diameter	4.04 ± 0.59	2.70	5.17
Pul./Ao	0.82 ± 0.10	0.66	1.35
Pul.Veloc. (cm/s)	91 ± 14.71	64.60	135.00
Pul.Gr. Pres.(mmHg)	3.39 ± 1.11	1.67	7.29
Aortic Veloc. (cm/s)	87.60 ± 14.95	60.30	130.60
Ao.Gr. Pres. (mmHg)	3.13 ± 1.06	1.45	6.82

The level of significance was defined as *p* < 0.05. IVSd: interventricular septal thickening in diastole; LVIDd: left ventricular internal diameter in diastole; LVFWd: left ventricular free wall thickness in diastole; IVSs: interventricular septal thickening in systole; LVIDs: left ventricular internal diameter in systole; LVFWs: left ventricular free wall thickness in systole; RIVDd: right ventricular diameter in diastole; EF: ejection fraction; FS: fractional shortening of the left ventricle; LA: left atrial diameter; Ao: internal diameter of the aortic root; LA/Ao: left atrium and aorta diameter ratio; Pul./Ao: pulmonary and aorta diameter ratio; Pul. Veloc.: Pulmonary velocity; Pres. Gr. Pul.: Pressure gradient between right ventricle and pulmonary artery; Aortic Veloc.: Aortic velocity; Ao. Pres. Gr.: Pressure gradient between the left ventricle and aorta.

**Table 3:** Electrocardiographic parameters in Pega breed donkeys (mean, standard deviation, minimum, maximum), males (*n* = 20) and females (*n* = 30).

Parameter	Mean ± SD	Minimum	Maximum
HR (bpm)	65.00 ± 16.50	35.00	95.00
P (ms)	111.00 ± 18.38	73.00	160.00
P (mV)	0.31 ± 0.05	0.18	0.46
PR (ms)	230.80 ± 41.57	150.00	337.00
QRS (ms)	112.36 ± 15.84	87.00	177.00
R (mV)	0.10 ± 0.02	0.04	0.20
S (mV)	1.90 ± 0.45	0.93	3.03
QT (ms)	432.74 ± 61.12	320.00	573.00
QTc (ms)	442.00 ± 32.90	349.00	538.00
T (ms)	137.58 ± 32.20	80.00	213.00
T (mV)	0.46 ± 0.17	0.23	0.90
RR (ms)	974.36 ± 277.47	630.00	1757.00

The level of significance was defined as *p* < 0.05.

## Data Availability

The datasets are available from the corresponding author on reasonable request.
